# *Terminalia catappa* Extract Palliates Redox Imbalance and Inflammation in Diabetic Rats by Upregulating Nrf-2 Gene

**DOI:** 10.1155/2021/9778486

**Published:** 2021-12-16

**Authors:** Franklyn Nonso Iheagwam, Gaber El-Saber Batiha, Olubanke Olujoke Ogunlana, Shalom Nwodo Chinedu

**Affiliations:** ^1^Department of Biochemistry, Covenant University, P.M.B. 1023 Ota, Ogun State, Nigeria; ^2^Covenant University Public Health and Wellbeing Research Cluster (CUPHWERC), Covenant University, P.M.B. 1023 Ota, Ogun State, Nigeria; ^3^Department of Pharmacology and Therapeutics, Faculty of Veterinary Medicine, Damanhour University, Damanhour 22511, AlBeheira, Egypt

## Abstract

This study aims at evaluating the ameliorative role of *Terminalia catappa* aqueous leaf extract (TCA) on hyperglycaemia-induced oxidative stress and inflammation in a high-fat, low dose streptozotocin-induced type 2 diabetic rat model. Experimental rats were treated orally with 400 and 800 mg/kg bw TCA daily for four weeks. Antioxidant enzyme activities, plasma glucose concentration, protein concentration, oxidative stress, and inflammation biomarkers were assayed using standard methods. Hepatic relative expressions of tumour necrosis factor-alpha (TNF-*α*), interleukin-six (IL-6), and nuclear factor-erythroid 2 related factor 2 (Nrf-2) were also assessed. Molecular docking and prediction of major TCA phytoconstituents' biological activity related to T2DM-induced oxidative stress were evaluated *in silico*. Induction of diabetes significantly (*p* < 0.05) reduced superoxide dismutase, glutathione-S-transferase, and peroxidase activities. Glutathione and protein stores were significantly (*p* < 0.05) depleted, while glucose, MDA, interleukin-six (IL-6), and tumour necrosis factor-*α* (TNF-*α*) concentrations were significantly (*p* < 0.05) increased. A significant (*p* < 0.05) upregulation of hepatic TNF-*α* and IL-6 expression and downregulation (*p* < 0.05) of Nrf-2 expression were observed during diabetes onset. TCA treatment significantly (*p* < 0.05) modulated systemic diabetic-induced oxidative stress and inflammation, mRNA expression dysregulation, and dysregulated macromolecule metabolism. However, only 800 mg/kg TCA treatment significantly (*p* < 0.05) downregulated hepatic TNF-*α* expression. 9-Oxabicyclo[3.3.1]nonane-2,6-diol and 1,2,3-Benzenetriol bound comparably to glibenclamide in Nrf-2, IL-6, and TNF-*α* binding pockets. They were predicted to be GST A and M substrate, JAK2 expression, ribulose-phosphate 3-epimerase, NADPH peroxidase, and glucose oxidase inhibitors. These results suggest that TCA ameliorates hyperglycaemia-induced oxidative stress and inflammation by activating Nrf-2 gene.

## 1. Introduction

Diabetes mellitus (DM) is a metabolic and endocrine disease of chronic nature which arises from a lack of insulin secretion, action, or both. It is characterised majorly by chronic hyperglycaemia, disturbance in the intermediary metabolism of major macromolecules (carbohydrate, protein, and lipid), and vascular complications arising from organ/system dysfunction [1, 2]. According to the International Diabetes Foundation (IDF), as of 2019, over 450 million adults were living with diabetes worldwide. This number is expected to increase by 2045 to about 700 million, with most of the diabetes risk and burden being type 2 diabetes mellitus (T2DM) [3]. There is a paradigm shift in Africa, in which the major focus of the continent's health system has moved from infectious diseases to noncommunicable diseases. This recent trend is due to increased urbanisation and lifestyle changes [4–6]. Irrespective of the paradigm shift, the use of medicinal plants in disease management has not changed. *Terminalia catappa* is one of such plants found in Nigeria with numerous pharmacological properties [7]. The leaf is a rich source of antioxidant and anti-inflammatory principles, notably flavonoids, phenols, and terpenes [1, 8].

The progression of many chronic diseases has been hinged on inflammation and oxidative stress interactions. Different studies have shown that, just like other noncommunicable diseases, oxidative stress plays a significant role in T2DM pathogenesis, progression, and complications [9–12]. Oxidative stress arises whenever the cellular redox balance is disrupted, leading to loss of biomolecular integrity and membrane damage. It promotes diabetes onset and aggravates this disorder and its associated complications by disrupting insulin release and action [13–15]. An increase in free radicals generates further oxidative stress by inducing mitochondria uncoupling, depleting the antioxidant defence system, and eliciting chronic inflammation by activating stress-activated kinases. Stimulation of proinflammatory cytokine and chemokine gene expression with subsequent increase in the circulation of these proinflammatory mediators results from stress kinase activation [16]. Dysfunction in glucose metabolism and chronic hyperglycaemia utilise oxidative stress and inflammatory mechanisms in the pathogenesis of diabetes-related complications [17]. Hyperglycaemia stimulates intra- and extracellular free radical generation inducing oxidative stress, concomitantly stimulating the generation of proinflammatory mediators [18]. It leads to the formation of toll-like receptors and inflammasome-complexes, which are key inducers for inflammation through nuclear factor-kappa B (NF-*κ*B) activation and oxidative stress progression [19]. Irregular epigenetic modifications and activation of interleukin-6 (IL-6), tumour necrosis factor-*α* (TNF-*α*), and NF-*κ*B genes with concomitant downregulation of superoxide dismutase 2 (SOD2), nuclear factor-erythroid 2 related factor 2 (Nrf-2), paraoxonase-1 (PON 1), and other antioxidant-related genes are also common features [20]. Damage of macromolecules is the downstream consequence of hyperglycaemic-induced oxidative stress, with the degree of damage associated with the duration of exposure [21].

Numerous pieces of information show that hyperglycaemia-induced oxidative stress is a major player in the pathogenesis and progression of T2DM and its related complications; hence, tackling it cannot be overemphasised [11, 17, 22–25]. With the increasing prevalence and incidence of T2DM in Nigeria and sub-Saharan Africa [2, 26], oxidative stress has become a focal point of interest in diabetic research [11]. Despite the reported antioxidant activities of oral antioxidant agents such as vitamins, their usefulness in preventing diabetic complications is elusive as human studies have shown that they do not decrease oxidative stress [27–29]. This observation has been attributed to their null effect on blood glucose levels [30]. Therefore, controlling oxidative stress and inflammation while maintaining glycaemic control is imperative in managing T2DM and preventing complications associated with diabetes. Medicinal plants and natural antioxidants with hypoglycaemic activity are hypothesised as a possible solution due to a plethora of phytochemicals and nonnutritional compounds [31]. Despite the reports on the antioxidant property of *T. catappa* in diabetic and nondiabetic animal models [32–35], information on the molecular mode of antioxidant and anti-inflammatory activity of *T. catappa* in T2DM animal model is lacking. This study evaluates the role of *Terminalia catappa* aqueous leaf extract in ameliorating hyperglycaemia-induced oxidative stress and inflammation in high-fat diet (HFD)/low-dose streptozotocin-induced diabetic rats. The extract's role on the expression of some genes relevant to redox status and inflammation was also evaluated.

## 2. Materials and Methods

### 2.1. Chemicals and Reagents

One-step RT-PCR kit (TransGen EasyScript®) was purchased from TransGen Biotech Co., Ltd. (Beijing, China). Glucose diagnostic kit was purchased from Randox Diagnostics (Ireland, UK). Streptozotocin (STZ), tumour necrosis factor-alpha (TNF-*α*), and interleukin-6 (IL-6) ELISA kits were products of Solarbio Science and Technology (Beijing, China). All other chemicals, unless stated otherwise, were purchased from Sigma-Aldrich, Germany.

### 2.2. Collection, Identification, and Preparation of Plants

Fresh mature leaves of *T. catappa* (TC) were acquired from Covenant University, Ota, Nigeria, and authenticated (FHI 112775) in Forest Research Institute of Nigeria (FRIN), Ibadan, Nigeria. The leaves were then air-dried and aqueous crude extract (TCA) was prepared as outlined by Iheagwam, Okeke [36]. Briefly, TC leaves were washed, air-dried under shade, and macerated in distilled water and the resulting filtrate was concentrated to dryness in a rotary evaporator to yield TCA.

### 2.3. Experimental Animals and Husbandry

Six-to-eight-week-old Male Wistar rats (*n* = 30) weighing 200 ± 20 g were used for this study. The animals were acclimatised for two weeks before the experiment, maintained under standard conditions (12 hrs light/dark cycle, room temperature (23 ± 2°C), humidity (50 ± 5%)), and provided food and water *ad libitum*. The experimental protocol was approved by Covenant University Health, Research and Ethics Committee (CHREC/031/2018), following institution guidelines for animal care.

### 2.4. Experimental Design

Rats (*n* = 30) were randomly divided into five [5] groups of 6 rats: Group I, normal rats treated with vehicle (distilled water) alone; Group II, diabetic rats treated with vehicle alone; Group III, diabetic rats treated with glibenclamide (10 mg/kg bw); Group IV, diabetic rats treated with 400 mg/kg bw of TCA; and Group V, diabetic rats treated with 800 mg/kg bw of TCA. The experimental period and dosage were based on a similar design utilised in the safety evaluation study of TCA [36, 37].

### 2.5. Diabetes Induction

T2DM was induced using a high-fat diet (HFD) that was self-formulated as shown in Table 1 and low-dose STZ (30 mg/kg bw) according to the method of Stalin et al. [38] with slight modification. Briefly, rats were fed with HFD to induce insulin resistance for eight weeks. Thereafter, DM was induced by intraperitoneal injection of STZ (30 mg/kg bw in citrate buffer [0.1 M, pH 4.5]) at 1 mL/kg bw. Five days after STZ injection, the tail vein of the rats was pricked and fasting blood glucose (FBG) was analysed. Rats with FBG > 200 mg/dL were considered diabetic and included in the study in which TCA treatment started on the 6th day after STZ induction. Administration of TCA, glibenclamide, and vehicle was done daily according to the experimental design by gastric intubation for 28 days. Fasting blood glucose levels and animal body weight were also recorded during the experiment. Rats were fasted for over 12 hours and thereafter anaesthetised using xylazine/ketamine (5 : 50 g/g) after the experimental period.

### 2.6. Sample Collection and Preparation

Blood was collected from the heart through cardiac puncture, placed in heparin, and separated to obtain plasma and erythrocytes [39]. Liver and kidney were excised, prepared, and stored as previously reported [36, 40].

### 2.7. Oxidative Stress and Biochemical Assays

Assessment of oxidative stress, inflammation, and other biochemical parameters was carried out in the plasma, erythrocytes, kidney, and liver. The activity of superoxide dismutase (SOD) was determined using the pyrogallol method of Marklund and Marklund [41], while the formation of 1-chloro-2,4-dinitrobenzene and glutathione conjugate was used to analyse glutathione-S-transferase (GST) activity according to the method of Habig et al. [42]. The concentration of reduced glutathione (GSH) was estimated using the method of Sedlak and Lindsay [43], while peroxidase (Px) activity was assayed following the method of Chance and Maehly [44]. The concentration of thiobarbituric reactive substances (MDA) was tested to determine lipid peroxidation as described by Buege and Aust [45]. Tumour necrosis factor-*α* (TNF-*α*) and interleukin-6 (IL-6) were analysed using Solarbio ELISA kits, while plasma blood glucose was assayed using Randox kit according to the manufacturers' instructions. Protein concentration was assayed according to the method of Lowry et al. [46].

### 2.8. RNA Extraction and Expression Analysis

Total hepatic RNA extraction using Trizol® reagent (TransGen Biotech Co., China) and reverse transcriptase-polymerase chain reaction (RT-PCR) analysis for TNF-*α*, IL-6, and nuclear factor-erythroid 2 related factor 2 (Nrf-2) were determined according to the methodology of Stalin et al. [38] with slight modification. RT-PCR was performed using TransScript® II One-Step RT-PCR kit (TransGen Biotech Co., China) and run in a C1000 thermal cycler (Bio-Rad, CA, USA) following set parameters:

- Initial denaturation at 95°C for 5 min;
- Denaturation at 95°C for 30 sec;
- Annealing at 51–53°C for 30 sec as indicated in Table 2 for each primer;
- Extension at 72°C for 30 sec;
- Final extension at 72°C for 7 min. (×40 cycles).

The gene-specific primers in Table 2 were used for first-strand cDNA synthesis, while GAPDH served as a reference. Ethidium bromide-stained agarose gel (1.5%) (Sigma Aldrich, Germany) was used to run the PCR products and viewed under UV light (UVP BioDoc-It™ Imaging System, Upland, CA, USA). Each experiment was repeated three times.

### 2.9. In Silico Analysis of TCA Major Phytochemicals

#### 2.9.1. Protein Modelling, Ligand Modelling, and Molecular Docking

In a previous report [1], 9-Oxabicyclo[3.3.1]nonane-2,6-diol and 1,2,3-Benzenetriol were identified as the major phytochemicals in TCA, accounting for 11.02% and 9.63%, respectively, of the total phytochemical constituents. Their structures were downloaded from PubChem and energy was minimised as reported by Iheagwam et al. [1]. The 3D crystal structures of human TNF-*α* and IL-6 were obtained from the RCSB protein data bank with PDB codes 5MU8 and 4O9H, respectively. There was no crystal structure of human Nrf-2 present in the RCSB protein data bank. The amino acid sequence of Nrf-2 (AAB32188.1) was obtained from NCBI for homology modelling using SWISS-MODEL. The sequence was queried using BLASTp and a suitable template was selected based on the target sequence, identity, and query coverage correlation. The modelled Nrf-2 was validated using the Ramachandran plot [47].

#### 2.9.2. Molecular Docking

Molecular docking was carried out according to the methodology of Iheagwam et al. [47]. AutoDock 4.2 was used to add Gasteiger charges and assign nonpolar hydrogen to ligands, selected diabetic drugs (metformin and glibenclamide), and protein targets (Nrf-2, TNF-*α*, and IL-6). AutoDock Vina was used to run the docking simulation of the prepared ligands and selected diabetic drugs in the active sites of the prepared proteins with the set grid map for each target at 0.375 Å, as shown in Table S1.

#### 2.9.3. Computational Activity and ADMET Prediction

The biological activity profile of 9-Oxabicyclo[3.3.1]nonane-2,6-diol and 1,2,3-Benzenetriol related to T2DM-induced oxidative stress was predicted using the prediction of activity spectra for substances- (PASS-) based approach [48]. The predicted activity of the compounds is estimated based on their structural formula identifying probable activity (Pa) and inactivity (Pi), which ranges from 0.000 to 1.000. However, only activities in which Pa > Pi and Pa > 0.7 were considered probable. The pharmacokinetics and toxicity properties of the compounds were predicted using SwissADME [49] and vNN-ADMET [50], respectively.

### 2.10. Data Analysis

Generated data were analysed using IBM Statistical Package for the Social Sciences v.23 (IBM Inc., New York, USA) and expressed as mean ± SEM of six biological replicates except otherwise stated. Statistical significance (*p* < 0.05) was determined using one-way analysis of variance (ANOVA) supplemented with Duncan multiple range test for post hoc analyses.

## 3. Results

### 3.1. T. Catappa Effect on HFD/STZ-Induced Weight Loss and Hyperglycaemia

**Figure 1:**
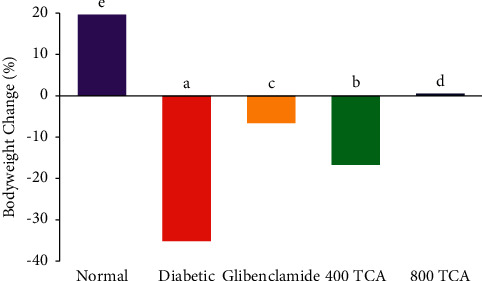
Effect of *T. catappa* aqueous extract treatment on bodyweight changes in HFD/STZ-induced diabetic rats. Bars represent proportion. Bars with different superscripts are significantly different at *p* < 0.05.

**Figure 2:**
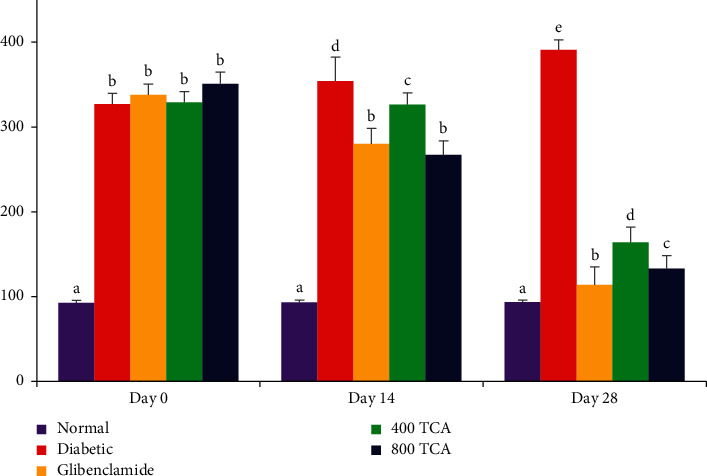
Effect of *T. catappa* aqueous extract treatment on fasting blood glucose in HFD/STZ-induced diabetic rats after 14 and 28 days of treatment. Bars represent mean ± SEM (*n* = 6). Bars with different superscripts are significantly different, while those with the same superscripts are not significantly different at *p* < 0.05.

In Figure 1, diabetes onset led to a significant (*p* < 0.05) weight loss of 35.2% in HFD/STZ-induced diabetic rats compared to 19.7% weight gain in normal rats at the end of the experiment. Administration of TCA significantly (*p* < 0.05) reduced the weight loss, with 0.5% weight gain recorded for 800 TCA administered group compared with glibenclamide (weight loss of 6.5%) and diabetic groups. Unlike animal weight, after diabetes induction, the fasting blood sugar was significantly (*p* < 0.05) raised in the diabetic rats compared with normal rats. Diabetic rats treated with TCA exhibited a dose-dependent decrease (*p* < 0.05) in fasting blood sugar after 14 and 28 days compared to untreated diabetic rats (Figure 2).

### 3.2. T. Catappa Ameliorates HFD/STZ-Induced Systemic Oxidative Stress

**Figure 3:**
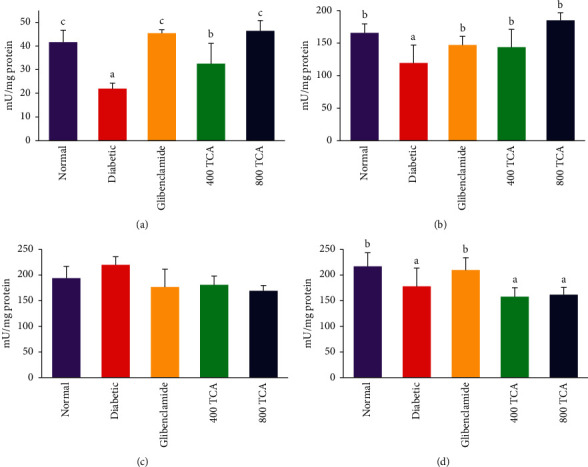
Effect of *T. catappa* aqueous extract treatment on (a) plasma, (b) hepatic, (c) renal, and (d) erythrocyte superoxide dismutase (SOD) activity in HFD/STZ-induced diabetic rats. Bars represent mean ± SEM (*n* = 6). Bars with different superscripts are significantly different, while those without superscripts are not significantly different at *p* < 0.05.

**Figure 4:**
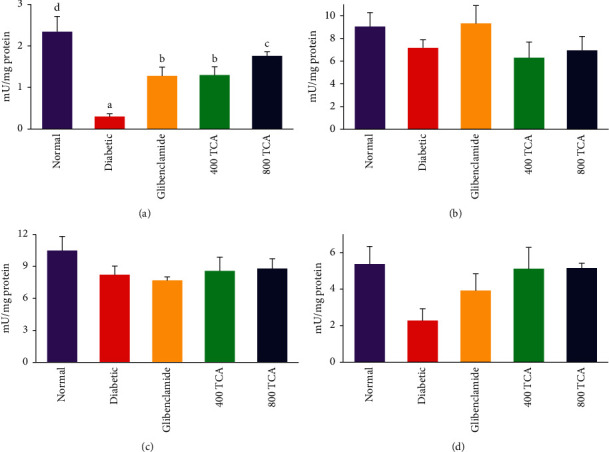
Effect of *T. catappa* aqueous extract treatment on (a) plasma, (b) hepatic, (c) renal, and (d) erythrocyte peroxidase (Px) activity in HFD/STZ-induced diabetic rats. Bars represent mean ± SEM (*n* = 6). Bars with different superscripts are significantly different, while those without superscripts are not significantly different at *p* < 0.05.

**Figure 5:**
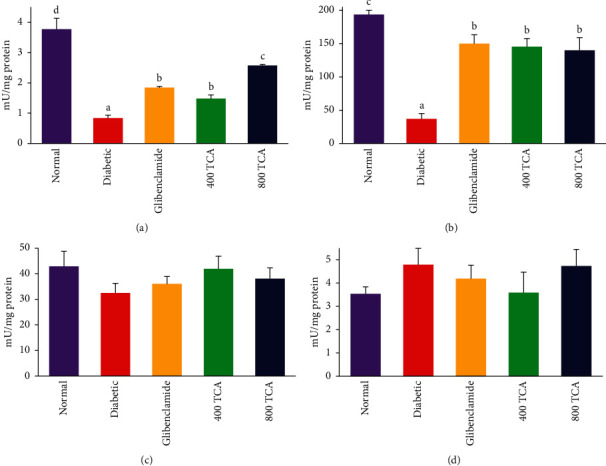
Effect of *T. catappa* aqueous extract treatment on (a) plasma, (b) hepatic, (c) renal, and (d) erythrocyte glutathione-S-transferase (GST) activity in HFD/STZ-induced diabetic rats. Bars represent mean ± SEM (*n* = 6). Bars with different superscripts are significantly different, while those without superscripts are not significantly different at *p* < 0.05.

The data for TCA treatment on enzymatic antioxidant activities are illustrated in Figures 3–5. In HFD/STZ-induced diabetic rats, there was a significant (*p* < 0.05) reduction in plasma, hepatic, and erythrocyte SOD activities by 47.1, 27.9, and 18.0% compared with normal rats. The plasma and hepatic SOD activities in diabetic rats treated with TCA were restored significantly (*p* < 0.05) to normal in a dose-dependent manner with a 110.9 and 54.8% respective increase recorded at the highest dose compared with the diabetic group. Nonetheless, the opposite was the case as TCA administration could not improve (*p* > 0.05) erythrocyte SOD activity at both doses compared with the diabetic group. There was no significant (*p* > 0.05) difference in renal SOD activity after diabetic insult and TCA treatment (Figure 3). Induction of diabetes did not significantly (*p* > 0.05) alter the activities of Px in the liver, kidney, and erythrocytes. However, the activity of plasma Px was significantly (*p* < 0.05) reduced by 87.2% in diabetic rats compared with normal rats. This observation was significantly (*p* < 0.05) improved upon by 333.3 and 486.7% in a dose-dependent manner after 400 and 800 mg/kg bw TCA treatment, respectively, compared with the diabetic group (Figure 4). In Figure 5, diabetes induction led to a significant (*p* < 0.05) reduction in plasma and hepatic GST activities by 77.7 and 80.9%, respectively, compared with normal rats. After 4-week TCA administration, plasma and hepatic GST activities were significantly (*p* < 0.05) increased in diabetic rats compared with the diabetic group. The observation was dose-dependent only in plasma GST activity as 76.2 and 206% increases were the respective ratios in 400 and 800 mg/kg bw TCA treatment. Induction of diabetes did not significantly (*p* > 0.05) alter the activities of GST in the kidney and erythrocytes (Figure 5).

**Figure 6:**
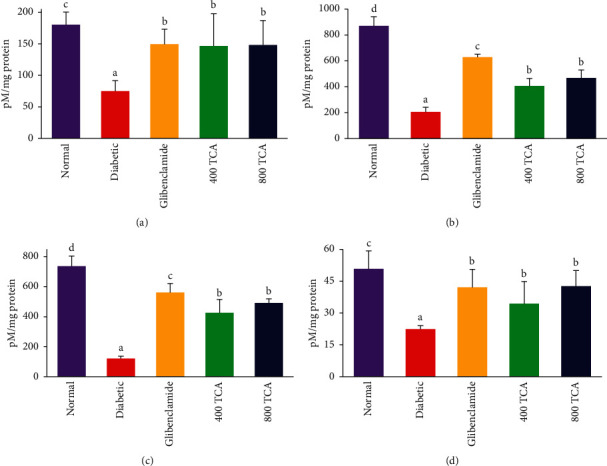
Effect of *T. catappa* aqueous extract treatment on (a) plasma, (b) hepatic, (c) renal, and (d) erythrocyte reduced glutathione (GSH) concentration in HFD/STZ-induced diabetic rats. Bars represent mean ± SEM (*n* = 6). Bars with different superscripts are significantly different at *p* < 0.05.

**Figure 7:**
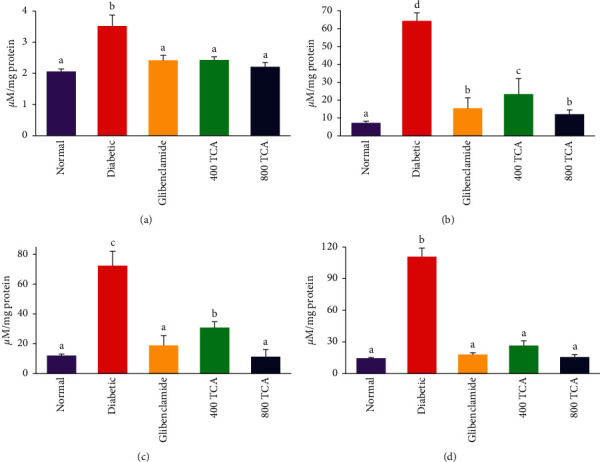
Effect of *T. catappa* aqueous extract treatment on (a) plasma, (b) hepatic, (c) renal, and (d) erythrocyte lipid peroxidation (MDA) concentrations in HFD/STZ-induced diabetic rats. Bars represent mean ± SEM (*n* = 6). Bars with different superscripts are significantly different at *p* < 0.05.

The data for TCA treatment on nonenzymatic antioxidant concentrations are illustrated in Figures 6 and 7. Administration of TCA at 400 and 800 mg/kg bw to diabetic rats significantly (*p* < 0.05) improved the plasma (95.4 and 97.4%, respectively), hepatic (97 and 127.1%, respectively), renal (254.1 and 308.6%, respectively), and erythrocytes (53.4 and 90.4%, respectively) GSH concentrations to near-normal levels when compared with the diabetic group. Plasma, hepatic, renal, and erythrocytes GSH concentrations were initially depleted significantly (*p* < 0.05) by 58.3, 76.3, 83.7, and 55.9%, respectively, after induction of diabetes when compared with normal rats (Figure 6). After diabetes induction, plasma, hepatic, renal, and erythrocyte MDA concentrations were significantly (*p* < 0.05) increased by 70.9, 784.6, 502.7, and 665.2%, respectively, compared with the normal rats. Upon 400 and 800 mg/kg bw TCA treatment, the MDA concentration of the diabetic rats was reduced significantly (*p* < 0.05). On the other hand, only renal and erythrocyte MDA levels of the rats were reduced to the normal level, while the reduction was dose-dependent in the kidney (57.5 and 84.4%, respectively) and liver (63.7 and 81.2%, respectively) (Figure 7).

### 3.3. T. Catappa Suppresses HFD/STZ-Induced Inflammation, Glucose, and Protein Dysregulation

Diabetic onset significantly (*p* < 0.05) elevated plasma glucose by 153.5% compared with normal rats. TCA 400 and 800 mg/kg bw treatment significantly reduced (*p* < 0.05) the elevated glucose concentration by 38.3 and 50.9%, respectively, in a dose-dependent manner when compared with the diabetic group. The reduction was not as significant as that observed for the drug group (55.4%). Induction of diabetes resulted in a significant (*p* < 0.05) increase in plasma and hepatic IL-6 (645.3 and 104.5%, respectively) and TNF-*α* (69.4 and 479.8%, respectively) concentrations when compared with normal rats. Nonetheless, there was no significant (*p* > 0.05) difference in renal IL-6 and TNF-*α* concentrations across all groups. Oral 400 and 800 mg/kg bw TCA treatment significantly (*p* < 0.05) decreased IL-6 and TNF-*α* concentrations in the liver by 53.4 and 69.1%, respectively, while 71.9 and 32.6% decreases were the respective ratios in the plasma when compared with diabetic rats. The inverse was the case for plasma and organ protein levels as they were significantly (*p* < 0.05) depleted after induction of diabetes compared with normal rats. However, upon treatment with TCA, these parameters were significantly (*p* < 0.05) improved compared to the untreated diabetic rats (Table 3).

### 3.4. T. Catappa Modulates HFD/STZ-Induced Dysregulation of Inflammatory mRNA Expression

**Figure 8:**
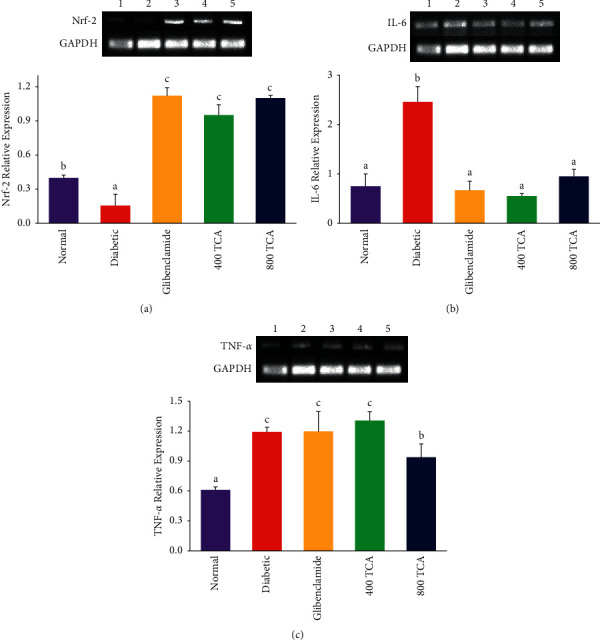
Effect of TCA on (a) Nrf-2, (b) IL-6, and (c) TNF-*α* mRNA expression in the liver of HFD/STZ-induced diabetic rats. Bars represent mean ± SEM (*n* = 3). Bars with different superscripts are significantly different at *p* < 0.05.

Induction of diabetes significantly (*p* < 0.05) reduced the expression of hepatic Nrf-2 mRNA in rats by 62.5% compared with the normal group. Treatment of diabetic rats with 400 and 800 mg/kg bw TCA significantly (*p* < 0.05) improved Nrf-2 expression by 533.3 and 633.3%, respectively, in the experimental group compared with the diabetic animals. The reverse was the case for hepatic IL-6 and TNF-*α*, as diabetes onset significantly (*p* < 0.05) increased the expression of these genes by 228.0 and 95.1%, respectively. Upon 400 and 800 mg/kg bw TCA treatment, IL-6 mRNA expression was significantly (*p* < 0.05) reduced by 77.6 and 65.4%, while only the highest treatment dosage (800 mg/kg) was able to reduce TNF-*α* mRNA expression by 21.0% when compared with the diabetic group (Figure 8).

### 3.5. Molecular Docking Study of Major T. Catappa Aqueous Leaf Extract Phytoconstituents

The selected template for the modelled Nrf-2 structure was 2LZ1 based on sequence identity (100%), sequence similarity (0.6), global model quality estimate (GMQE = 0.05), QMEANDisCo score (0.75 ± 0.11), oligomeric state (monomer), and experimental comparison plot superiority over other templates. In the modelled Nrf-2 structure, 89.2, 10.6, and 1.4% of the amino acid residues were in the most favoured, additional allowed, and disallowed region, respectively (Figure S1).

In Table 4, 9-Oxabicyclo[3.3.1]nonane-2,6-diol (−4.5, −4.7, and −5.0 kcal/mol, respectively) and 1,2,3-Benzenetriol (−4.3, −5.3, and −5.6 kcal/mol, respectively) exhibited better binding capacity than metformin (−4.2, −4.6, and −4.3 kcal/mol, respectively) in the pocket of Nrf-2, IL-6, and TNF-*α*. However, glibenclamide (−7.2, −7.6, and −6.4 kcal/mol, respectively) exhibited the best binding affinity for the three protein targets. For the binding interactions, all ligands were stabilised by hydrogen bonds, pi bonds, and Van der Waals interactions with amino acid residues in the binding site. Unlike other ligands, metformin did not form a pi bond in the binding pocket of the targets but rather distinct interactions such as salt-bridge interaction and attractive bond with Nrf-2 (Glu442) and IL-6 (Glu42), respectively. Pro453 was the only amino acid residue in the binding pocket involved in the stabilising of all ligands in the binding pocket of Nrf-2, while numerous residues were observed for IL-6 (Leu39, Glu42, Thr43, Leu101, Arg104, Ala112, Thr163, and Leu167) and TNF-*α* (Val17, Ala18, Pro20, Phe144, Ser147, Gly148, Gln149, and Val150) (Table 4). The binding conformations of all ligands in the binding pockets of Nrf-2, IL-6, and TNF-*α* are shown in Figures S2–S4, respectively.

### 3.6. Predicted Bioactivity and ADMET Properties of Major T. Catappa Aqueous Leaf Extract Phytoconstituents

For the predicted bioactivity, 9-Oxabicyclo[3.3.1]nonane-2,6-diol and 1,2,3-Benzenetriol were predicted to be GST A and M substrates, JAK2 expression, ribulose-phosphate 3-epimerase, NADPH peroxidase, and glucose oxidase inhibitors. HIF1A expression and bisphosphoglycerate phosphatase inhibition were peculiar to 9-Oxabicyclo[3.3.1]nonane-2,6-diol and 1,2,3-Benzenetriol, respectively (Table 5). From Table 6, 9-Oxabicyclo[3.3.1]nonane-2,6-diol and 1,2,3-Benzenetriol were predicted to be very soluble, easily absorbed in the human intestine, bioavailable, easily synthesized, and stable in the hepatic microsome. They were also not predicted to be P-glycoprotein substrate and inhibitors, violators of Lipinski, Veber and Egan rule of drug-likeness, and cytochrome P_450_ isoforms inhibitor. These ligands were predicted to be unable to cause cardiotoxicity, drug-induced liver injury, cytotoxicity, mitochondrial toxicity, and mutagenicity. However, 1,2,3-Benzenetriol was predicted to easily permeate the blood-brain barrier, inhibit CYP3A4, and cause mitochondrial toxicity. 384 and 90 mg/day were the predicted maximum recommended therapeutic doses for 9-Oxabicyclo[3.3.1]nonane-2,6-diol and 1,2,3-Benzenetriol, respectively (Table 6).

## 4. Discussion

The significant weight loss observed in the diabetic rats may be due to poor glycaemic control. This major anthropometric observation shifts the energy demand to fat and protein stores. Various studies have reported similar findings on the weight reduction in diabetic rats buttressing this study [51–53]. The modulation of weight loss by TCA may be ascribed to the improvement of glycaemic control. The onset of hyperglycaemia in the untreated diabetic rats could result from poor utilisation of circulating glucose as an energy source by GLUT-4-dependent tissues [54]. The hypoglycaemic activity of TCA in diabetic rats, especially at the highest dose, might indicate improvement in the utilisation of glucose by peripheral tissues, further corroborating the modulatory effect of TCA on HFD/STZ-induced weight loss.

In diabetes progression, there is a *de novo* generation of reactive species, which promotes oxidative stress [55]. In conditions where chronic hyperglycaemia occurs, these generated reactive species further suppress the antioxidant defence systems in various tissues, exacerbating oxidative stress to maintain T2DM progression and complication development [56]. Enzymatic (SOD, Px, and GST) and nonenzymatic antioxidants (GSH and vitamins E and C) are endogenous biomolecules that scavenge free radicals preventing oxidative stress onslaught in cells. SOD scavenges superoxide radicals via enzymatic conversion to hydrogen peroxide, which GPx and catalase then catalyse to yield water and oxygen. The oxidative stress markers were changed in the rats as a result of the onset of diabetes. GSH concentration, SOD, GST, and Px activities were reduced with a concomitant increase in MDA concentration, reflecting impaired antioxidant defence with a subsequent increase in oxidative stress [57, 58]. However, TCA treatment improved the impaired antioxidant system while reducing oxidative stress (Figures 1–5). Previous studies corroborated these findings on the antioxidant effect of plant extracts and isolated natural compounds on HFD/STZ-induced diabetic rats [59, 60]. The increased hepatic GST activity could be ascribed to the phytochemicals present in TCA. Their biological activity prediction as GST substrates further lents credence to the findings. The reduction in the activities of the antioxidant enzymes in the diabetic group could be attributed to an increase in free radicals, glycosylation of enzymes, and loss of cofactors [61]. The deleterious effect of diabetes on SOD activity and lipid peroxidation impacted the erythrocytes more than other tissues. The observed high susceptibility of the erythrocyte to oxidative stress may be due to molecular oxygen, ferrous ions, and polyunsaturated fatty acids presence [16]. In addition, due to the absence of a nucleus and other cell organelles, there is limited repair capacity on oxidative stress-related damage [62]. The absence of these organelles could be the reason why TCA was unable to increase the reduced activity of SOD in the erythrocyte. The inhibition of ATP production by glycation products may have been responsible for the depletion of the erythrocyte GSH level [63]. These findings were similar to a previous report in alloxan-induced diabetic rats [64]. The inability of diabetic onset to affect renal enzymatic antioxidants might be due to the length of experimental duration, which was not sufficient to cause this complication, with nephropathy reported to occur gradually [65].

The observed improvement of the impaired antioxidant system of the organs and plasma might also be ascribed to the upregulation of Nrf-2 expression and proinflammatory gene downregulation (Figure 5). Nrf-2 is a transcription factor that induces phase II detoxifying and antioxidant enzymes, playing an important role in the antioxidant response, glucose metabolism, and prevention of DM onset [66–68]. It has also been reported to interact with Kelch-like ECH-associated protein 1 (Keap1) to protect organs/tissues of diabetics against diabetes-induced organopathy [69]. The observed increase in the concentration of these inflammatory mediators (IL-6 and TNF-*α*) after diabetes induction in rats was suppressed by TCA treatment, suggesting the anti-inflammatory effect of TCA in diabetic rats (Table 3). Proinflammatory cytokines are associated with diabetes progression through subclinical chronic inflammation, an independent risk factor for T2DM development [59]. Impaired hepatocyte insulin signalling and inhibition of insulin secretion from the *β*-cells of the pancreas after glucose stimulation is associated with elevated IL-6 [59]. During excessive ROS generation, activated NF*κ*B activates the release of other proinflammatory cytokines, instigating further ROS generation and establishing oxidative stress in the process [70, 71]. These inflammatory cytokines can interfere with insulin receptors on tissues and their signalling pathways, leading to complications such as insulin resistance [72]. Insulin resistance is directly associated with an increase in inflammatory biomarker concentration. These inflammatory mediators alter *β*-cell function through direct action or other secondary pathways to maintain T2DM onset [16]. Upregulation of proinflammatory cytokines mRNA would increase the level of tissue and systemic circulating levels of these cytokines. They are well documented to be associated with dysregulating downstream processes in the insulin signalling pathway [73, 74]. TCA's downregulation of these inflammatory genes may have resulted in reversing the dysregulation of insulin signalling, concomitantly reducing hyperglycaemia and its resultant oxidative and inflammatory effects. This probable mechanism has been postulated by a previous study [75]. In addition, the synthesis of proteins could be realised as one of the downstream effects of insulin signalling dysregulation, repair, and reversal. It is noteworthy that systemic protein levels are affected due to a defect in glucose metabolism as protein store impairment is a consequence of diabetes [76]. Proper utilisation of plasma glucose as a source of energy in TCA treatment groups may have resulted in suppressing protein store dysregulation, making it available for the synthesis of antioxidant enzymes and glutathione.

The binding conformations of the ligands in Nrf-2, IL-6, and TNF-*α* active sites suggest that TCA phytoconstituents and glibenclamide interacted with these proteins in their active site. Thus, TCA may probably ameliorate oxidative stress and inflammation via a similar mechanism as glibenclamide [38]. Also, the allosteric binding of 9-Oxabicyclo[3.3.1]nonane-2,6-diol in IL-6 active pocket may hint at TCA's probable additional anti-inflammatory mechanism. A study on the network pharmacology of andrographolide reported similar allosteric binding and interactive amino acid residues in the binding pocket of IL-6 [77]. A study on the possible activation of Nrf-2 by vitamin E and curcumin by Mishra et al. [78] reported a different binding conformation to our study. This observation could result from the size of their ligands, hence the preference for the binding pocket located at the c-terminal. The better binding affinity exhibited by glibenclamide over metformin on proteins associated with hyperglycaemia-induced oxidative stress and inflammation buttresses the choice of hypoglycaemic agent in this study.

The predicted inhibition of Janus kinase-2 (JAK2) expression, hypoxia-inducible factor-1*α* (HIF1A) expression, and NADPH peroxidase would suggest possible mechanisms through which TCA might alleviate glucose-induced toxicity (Table 4). Oxidative stress is known to be closely associated with activation or overexpression of NADPH peroxidase, while JAK2 expression enhances reactive species and proinflammatory cytokine generation [79]. Inhibition of JAK2 and HIF1A expression can partially attenuate hyperglycaemia-elicited oxidative stress [80, 81]. Glucose oxidase catalyses glucose conversion to gluconolactone and subsequently to glucuronic acid and H_2_O_2_ [82]. Ribulose-phosphate 3-epimerase and bisphosphoglycerate phosphatase are enzymes found in the pentose phosphate pathway and the glycolytic pathway. During diabetes onset, these enzymes catalyse reactions that give rise to increased glyceraldehyde-3-phosphate, which is subsequently converted to methylglyoxal inducing free radical generation, proinflammation, and oxidative stress [83]. Predicted inhibition of these enzymes by TCA phytochemicals may be potential mechanisms of tackling oxidative stress in the diabetic state. Pa was >0.7, suggesting that the pharmacological action experimental validation would probably be similar to the predicted results. The predicted mitochondria toxicity of 1,2,3-Benzenetriol might be due to its ability to generate H_2_O_2_. This toxic effect is usually counteracted by reduction of therapeutic dose as predicted and use of other natural antioxidants [84]. Irrespectively, the predicted ADMET properties and obedience of 9-Oxabicyclo[3.3.1]nonane-2,6-diol and 1,2,3-Benzenetriol to the drug-likeness rules would suggest these compounds as potential leads in controlling oxidative stress and inflammation while maintaining glycaemic control.

## 5. Conclusion

In conclusion, the aqueous extract of *T. catappa* (TCA) leaves possesses significant antioxidant and anti-inflammatory effects, which may be ascribed to the upregulation of the Nrf-2 gene and downregulation of inflammatory cytokine genes. These pharmacological activities are reported for the first time and may help slow down and overcome DM-related complications associated with hyperglycaemia-induced oxidative stress and inflammation. The inability to investigate the effect of TCA on glucose metabolism and Nrf-2 signalling pathway elements was a limitation in this study which requires further research. Further studies can be carried out to isolate these antioxidant principles from *T. catappa* leaves and experimentally validate their *in silico* prediction results as possible antioxidant mechanisms of TCA in T2DM.

## Figures and Tables

**Table 1:** Normal and high-fat diet chow formulation.

Feed composition	Weight (g/kg)	
	Normal diet	High-fat diet
Maize	450	50
Beef tallow	0	400
Groundnut cake	100	100
Flour binder	100	100
Soybean meal	50	50
Full fat soya	50	50
Palm kernel cake	50	50
Fish meal	100	100
Wheat offal	50	50
Bone	10	10
Premix^*∗*^	30	30
Methionine	6	6
Lysine	4	4

^*∗*^Contained (per kg): vitamin A (4 000 000 IU), vitamin D3 (800 000 IU), vitamin E (8 000 IU), vitamin K_3_ (0.9 g), thiamine (0.7 g), riboflavine (2 g), pyridoxine (1.2 g), vitamin B12 (0.006 g), nicotinic acid (11 g), pantothenic acid (3 g), folic acid (3 g), biotin (0.02 g), choline (120 g), CuSO_4_·5H_2_O (2 g), CoCl_2_·6H_2_O (0.008), NaCl (2 g), FeSO_4_·7H_2_O (8 g), KI (0.48 g), MnSO_4_·7H_2_O (32 g), CaSO_4_ (14 g), and ZnSO_4_ (20 g).

**Table 2:** Gene-specific primer sequence.

Gene	Sequence (5′-3′)	Annealing temperature (°C)
IL-6	5′-ATTGTATGAACAGCGATGATGCAC-3′ (F)	51
	5′-CCAGGTAGAAACGGAACTCCAGA-3′ (R)	
Nrf-2	5′-GGGCAAAAGCTCTCCATATTCC-3′ (F)	52
	5′-GAGCGGCAACTTTATTCTTCCC-3′ (R)	
TNF-*α*	5′-ACGGCATGGATCTCAAAGAC-3′ (F)	53
	5′-CGGACTCCGCAAAGTCTAAG-3′ (R)	
GAPDH	5′-CTGACATGCCGCCTGAAAC-3′ (F)	51
	5′-CCAGCATCAAAGGTGGAAGAA-3′ (R)	

**Table 3:** Effect of *T. catappa* aqueous extract treatment on other biochemical parameters in HFD/STZ-induced diabetic rats.

	Normal	Diabetic	Glibenclamide	400 TCA	800 TCA
Plasma glucose (mg/dL)	101.42 ± 14.10^a^	257.06 ± 15.04^d^	114.62 ± 13.13^a^	158.51 ± 11.42^c^	126.32 ± 14.51^b^

TNF-*α* (pg/mL)					
Plasma	9.37 ± 1.56^a^	15.87 ± 1.58^d^	11.27 ± 1.60^c^	12.66 ± 3.70^c^	10.69 ± 1.11^b^
Liver	3.37 ± 0.65^a^	19.54 ± 1.40^c^	11.83 ± 22.10^b^	10.21 ± 16.86^b^	6.03 ± 10.98^a^
Kidney	9.92 ± 1.36	9.39 ± 2.67	9.91 ± 1.88	10.33 ± 1.01	8.47 ± 1.73

IL-6 (pg/mL)					
Plasma	28.38 ± 6.77^a^	211.52 ± 27.00^e^	94.22 ± 8.06^c^	147.05 ± 14.97^d^	59.39 ± 10.28^b^
Liver	7.75 ± 0.88^a^	15.85 ± 1.12^e^	13.99 ± 4.62^d^	9.36 ± 1.78^c^	7.39 ± 1.41^b^
Kidney	5.36 ± 0.32	7.74 ± 0.85	6.45 ± 1.87	6.35 ± 0.36	5.91 ± 1.49

Total protein (mg/mL)					
Plasma	539.60 ± 14.09^c^	463.49 ± 2.07^a^	501.81 ± 13.75^b^	509.76 ± 13.59^b^	532.23 ± 13.16^c^
Liver	154.33 ± 13.49^c^	109.35 ± 3.18^a^	138.45 ± 4.22^b^	132.31 ± 5.04^b^	137.11 ± 4.99^b^
Kidney	124.99 ± 8.12^d^	99.55 ± 1.49^a^	120.39 ± 0.91^c^	109.80 ± 1.90^b^	125.41 ± 0.93^d^
Erythrocyte	133.60 ± 6.38^c^	95.08 ± 2.85^a^	127.62 ± 10.91^b^	123.33 ± 4.24^b^	135.71 ± 3.05^c^

Data are represented as mean ± SEM (*n* = 6). Values with different superscripts across a row are significantly different, while those without superscripts are not significantly different at *p* < 0.05.

**Table 4:** Molecular docking of hypoglycaemic agents and major TCA phytoconstituents in the binding sites of Nrf-2, IL-6, and TNF-*α* proteins.

Compound	BA^*∗*^	H-Bond	Π-Bond	VdW Interactions	Others
Nrf2					
9-Oxabicyclo[3.3.1]nonane-2,6-diol	−4.5	Arg501	Lys500, Arg501, Ala495	Pro453, Gln496, Asn497, Arg499	Cys498^a^
1,2,3-Benzenetriol	−4.3	His437	Val454	Glu435, Leu438, Thr439, Glu442, Pro453, Glu455	Arg433^a^
Glibenclamide	−7.2	Asn505, Arg501^b^	Lys500, Leu503, Pro453, Val454	Glu435, Leu438, Glu442, Ala445, Lys446, Ile450, Phe452, Glu455, Val507	—
Metformin	−4.2	Glu435, His437	—	Arg433, Leu438, Thr439, Pro453, Val454, Glu455, Arg501, Leu503, Asn505	Glu442^c^

IL-6					
9-Oxabicyclo[3.3.1]nonane-2,6-diol	−4.7	Glu99, Asn144	Val96, Pro141	Lue92, Glu95, Pro139, Leu148	—
1,2,3-Benzenetriol	−5.3	Leu39, Ser108	Leu39, Leu101, Ala112	Glu42, Thr43, Arg104, Thr163, Leu167	—
Glibenclamide	−7.6	Thr43, Arg104, Gln156	Leu39, Tyr100, Leu101, Ala112	Glu42, Lys46, Asn103, Ser107, Gln152, Gln159, Asp160, Thr163, Leu167	—
Metformin	−4.6	Leu39, Glu42^b^, Thr43^b^, Arg104	—	Arg40, Leu101, Ser107, Ser108, Ala112, Thr163, Leu167	Glu42^d^

TNF-*α*					
9-Oxabicyclo[3.3.1]nonane-2,6-diol	−5.0	Ala145, Glu146, Ser147^b^	Val17, Ala18, Pro20, Val150	Asp143, Phe144, Gly148, Gln149	—
1,2,3-Benzenetriol	−5.6	Ala18, Gly148	Ala18, Pro20, Val150	Val17, Phe144, Ala145, Ser147, Gln149, Tyr151	Val150^a^, Glu146^a^
Glibenclamide	−6.4	Glu146, Gly148, Gln149, Val150	Val17, Pro20	Ala18, Leu29, Arg32, Ala33, Asn34, Phe144, Ala145, Ser147	—
Metformin	−4.3	Ala18, Asn34, Ser147^b^, Gly148	—	Val17, Pro20, Leu29, Phe144, Gln149, Val150	—

^*∗*^kcal/mol, ^a^unfavourable bonds, ^b^carbon-hydrogen bonds, ^c^salt-bridge interaction, ^d^attractive bond, BA: binding affinity, H: conventional hydrogen, VdW: Van der Waals.

**Table 5:** PASS-predicted T2DM-induced oxidative stress activity of major *T. catappa* aqueous leaf extract phytoconstituents.

Pa	Pi	Activity
9-Oxabicyclo[3.3.1]nonane-2,6-diol		
0.881	0.003	Ribulose-phosphate 3-epimerase inhibitor
0.855	0.008	HIF1A expression inhibitor
0.809	0.008	JAK2 expression inhibitor
0.802	0.009	GST A substrate
0.798	0.012	NADPH peroxidase inhibitor
0.768	0.016	Glucose oxidase inhibitor

1,2,3-Benzenetriol		
0.907	0.003	NADPH peroxidase inhibitor
0.898	0.003	JAK2 expression inhibitor
0.892	0.004	Glucose oxidase inhibitor
0.858	0.004	Ribulose-phosphate 3-epimerase inhibitor
0.854	0.004	Bisphosphoglycerate phosphatase inhibitor
0.787	0.011	GST A substrate
0.711	0.004	GST M substrate

**Table 6:** Predicted ADMET properties of major *T. catappa* aqueous leaf extract phytoconstituents and standard diabetic drugs.

	Absorption and distribution						Drug-likeness				
	Solubility class	HIA	BBB	Pgp-S	Pgp-I	LV	VV	EV	B	SA	MRTD^*∗*^
9-Oxabicyclo[3.3.1]nonane-2,6-diol	Very	High	No	No	No	0	0	0	0.55	4.31	384
1,2,3-Benzenetriol	Very	High	Yes	No	No	0	0	0	0.55	1	90
Glibenclamide	Poor	Low	No	No	Yes	1	1	0	0.55	3.42	183
Metformin	High	High	No	No	No	0	0	0	0.55	3.02	3000

	Metabolism							Toxicity			
	HLM	Cytochrome P_450_ inhibitor					hERG	DILI	HepG2	MMP	Ames test
		1A2	2C19	2C9	2D6	3A4					
9-Oxabicyclo[3.3.1]nonane-2,6-diol	Yes	No	No	No	No	No	No	No	No	No	No
1,2,3-Benzenetriol	Yes	No	No	No	No	Yes	No	No	No	Yes	No
Glibenclamide	No	No	Yes	Yes	No	Yes	No	Yes	No	No	No
Metformin	Yes	No	No	No	No	No	No	No	No	No	No

^*∗*^mg/day, HIA: human intestinal absorption, BBB: blood-brain barrier permeation, Pgp-S: P-glycoprotein substrate, Pgp-I: P-glycoprotein inhibitor, LV: Lipinski violation, VV: Veber violation, EV: Egan violation, B: bioavailability, SA: synthetic accessibility, MRTD: maximum recommended therapeutic dose, HLM: human liver microsomal stability, hERG: human ether-à-go-go-related gene, DILI: drug-induced liver injury, HepG2: HepG2 cytotoxicity, MMP: mitochondrial membrane potential.

## Data Availability

All the data are included in the manuscript and supplementary text.

## Supplementary Materials

Supplementary Materials Table S1: grid map specifications for molecular docking. Figure S1: representation of (a) modelled Nrf-2 structure and (b) corresponding Ramachandran plot. Figure S2: 2D representation of (a) 9-Oxabicyclo[3.3.1]nonane-2,6-diol, (b) 1,2,3-Benzenetriol, (c) glibenclamide, and (d) metformin interactions in the binding pocket of Nrf-2. Green, light blue, pink, orange, violet, and broken red lines represent conventional hydrogen, carbon-hydrogen, pi-alkyl, salt bridge, pi-sigma, and unfavourable bonds, respectively. Light green circles represent Van der Waals interactions. Figure S3: 2D representation of (a) 9-Oxabicyclo[3.3.1]nonane-2,6-diol, (b) 1,2,3-Benzenetriol, (c) glibenclamide, and (d) metformin interactions in the binding pocket of IL-6. Green, light blue, pink, orange, and violet broken lines represent conventional hydrogen, carbon-hydrogen, pi-alkyl, attractive, and pi-sigma bonds, respectively. Light green circles represent Van der Waals interactions. Figure S4: 2D representation of (a) 9-Oxabicyclo[3.3.1]nonane-2,6-diol, (b) 1,2,3-Benzenetriol, (c) glibenclamide, and (d) metformin interactions in the binding pocket of TNF-*α*. Green, light blue, pink, violet, and broken red lines represent conventional hydrogen, carbon-hydrogen, pi-alkyl, pi-sigma, and unfavourable bonds, respectively. Light green circles represent Van der Waals interactions.
