# Supplementary material for: Terminalia catappa Extract Palliates Redox Imbalance and Inflammation in Diabetic Rats by Upregulating Nrf-2 Gene
Source: Int J Inflam. 2021 Dec 16;2021:9778486. doi: 10.1155/2021/9778486 (PMC8702315; doi:10.1155/2021/9778486)
Supplement: Supplementary Materials — Table S1: grid map specifications for molecular docking. Figure S1: representation of (a) modelled Nrf-2 structure and (b) corresponding Ramachandran plot. Figure S2: 2D representation of (a) 9-Oxabicyclo[3.3.1]nonane-2,6-diol, (b) 1,2,3-Benzenetriol, (c) glibenclamide, and (d) metformin interactions in the binding pocket of Nrf-2. Green, light blue, pink, orange, violet, and broken red lines represent conventional hydrogen, carbon-hydrogen, pi-alkyl, salt bridge, pi-sigma, and unfavourable bonds, respectively. Light green circles represent Van der Waals interactions. Figure S3: 2D representation of (a) 9-Oxabicyclo[3.3.1]nonane-2,6-diol, (b) 1,2,3-Benzenetriol, (c) glibenclamide, and (d) metformin interactions in the binding pocket of IL-6. Green, light blue, pink, orange, and violet broken lines represent conventional hydrogen, carbon-hydrogen, pi-alkyl, attractive, and pi-sigma bonds, respectively. Light green circles represent Van der Waals interactions. Figure S4: 2D representation of (a) 9-Oxabicyclo[3.3.1]nonane-2,6-diol, (b) 1,2,3-Benzenetriol, (c) glibenclamide, and (d) metformin interactions in the binding pocket of TNF-α. Green, light blue, pink, violet, and broken red lines represent conventional hydrogen, carbon-hydrogen, pi-alkyl, pi-sigma, and unfavourable bonds, respectively. Light green circles represent Van der Waals interactions. [file 9778486.f1.docx]

***Terminalia catappa* extract palliates redox imbalance and inflammation in diabetic rats by upregulating Nrf-2**

**Franklyn Nonso Iheagwam^1,2*^, Gaber El-Saber Batiha^3^, Olubanke Olujoke Ogunlana^1,2^ and Shalom Nwodo Chinedu^1,2^**

^1^Department of Biochemistry, Covenant University, P.M.B. 1023 Ota, Ogun State, Nigeria.

^2^Covenant University Public Health and Wellbeing Research Cluster (CUPHWERC), Covenant University, P.M.B. 1023 Ota, Ogun State, Nigeria.

^3^Department of Pharmacology and Therapeutics, Faculty of Veterinary Medicine, Damanhour University, Damanhour 22511, AlBeheira, Egypt.

*Corresponding Author: [franklyn.iheagwam@covenantuniversity.edu.ng](mailto:franklyn.iheagwam@covenantuniversity.edu.ng)

**Table S1:** Grid map specifications for molecular docking

|  |  | **Nrf-2** | **IL-6** | **TNF-α** |
| --- | --- | --- | --- | --- |
|  | **x** | -1.19 | 34.91 | 15.04 |
| **Centre** | **y** | -0.87 | 23.38 | -6.24 |
|  | **z** | 5.38 | 11.08 | 34.67 |
|  | **x** | 37.53 | 38.14 | 41.95 |
| **Dimension** | **y** | 36.59 | 39.37 | 41.85 |
|  | **z** | 38.20 | 49.98 | 58.78 |

| **a)** 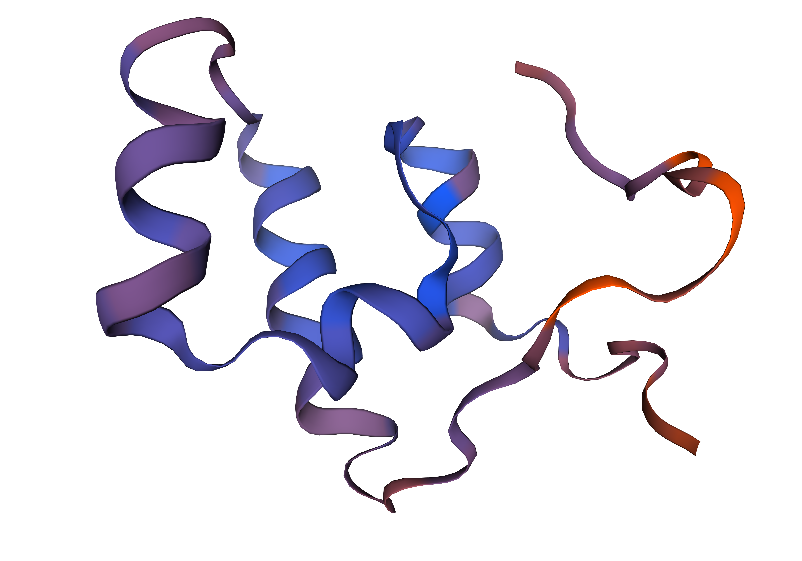 | **b)** 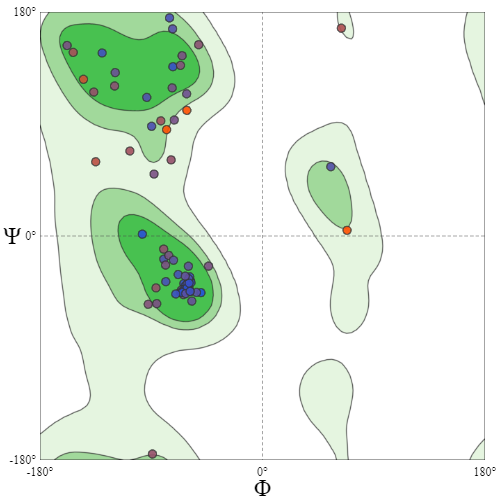 |
| --- | --- |

**Figure S1:** Representation of (a) Modelled Nrf-2 structure and (b) corresponding Ramachandran plot.

| **a) 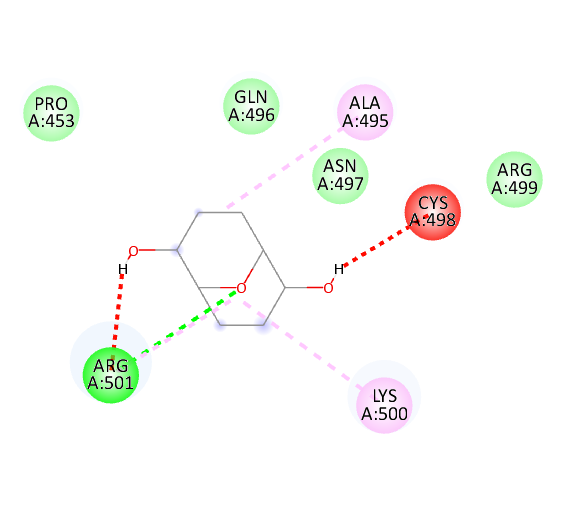** | **b) 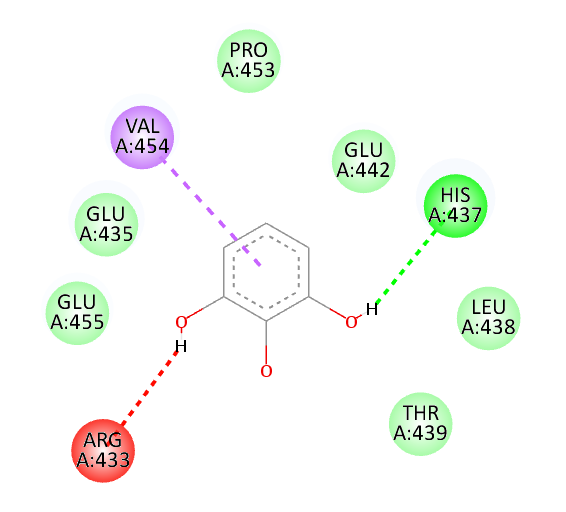** |
| --- | --- |
| **c) 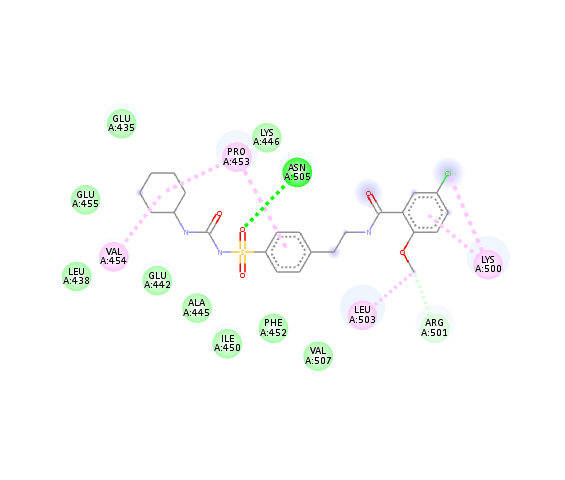** | **d) 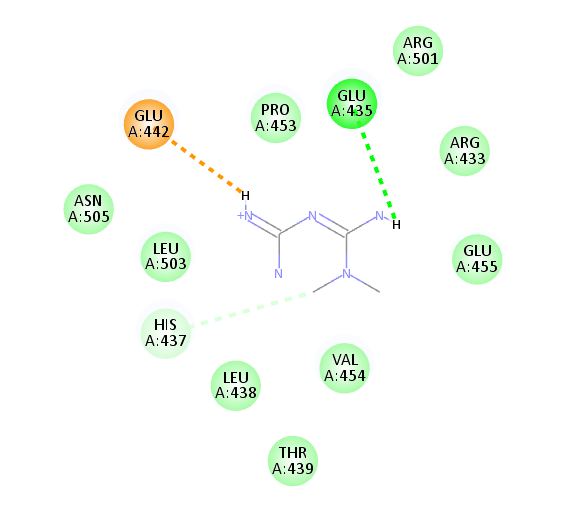** |

**Figure S2:** 2D representation of (a) 9-Oxabicyclo[3.3.1]nonane-2,6-diol, (b) 1,2,3-Benzenetriol, (c) Glibenclamide and (d) Metformin interactions in the binding pocket of Nrf-2. Green, light blue, pink, orange, violet and broken red lines represent conventional hydrogen, carbon-hydrogen, pi-alkyl, salt bridge, pi-sigma and unfavourable bonds, respectively. Light green circles represent Van der Waals interactions.

| **a) 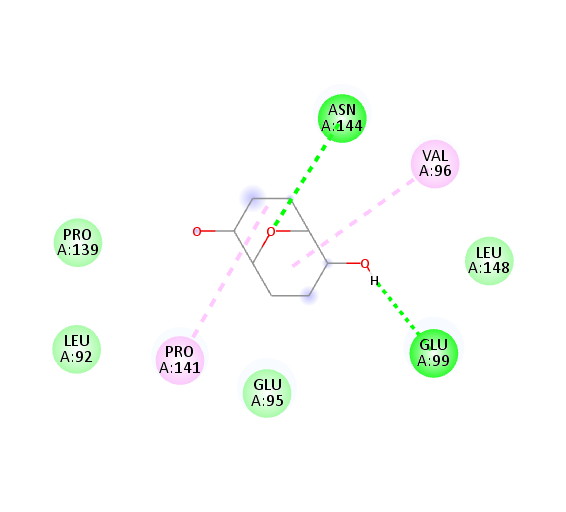** | **b) 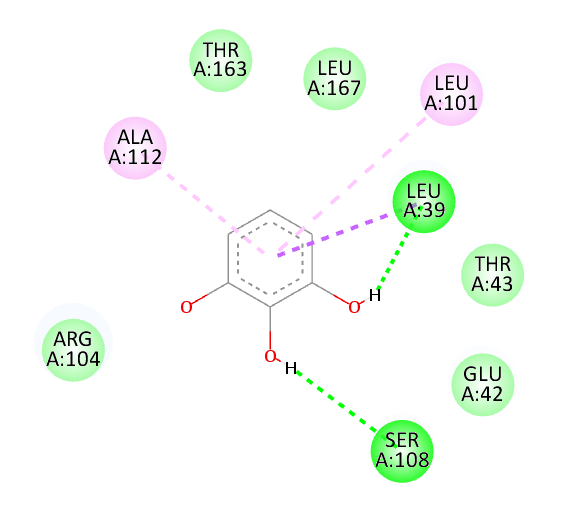** |
| --- | --- |
| **c) 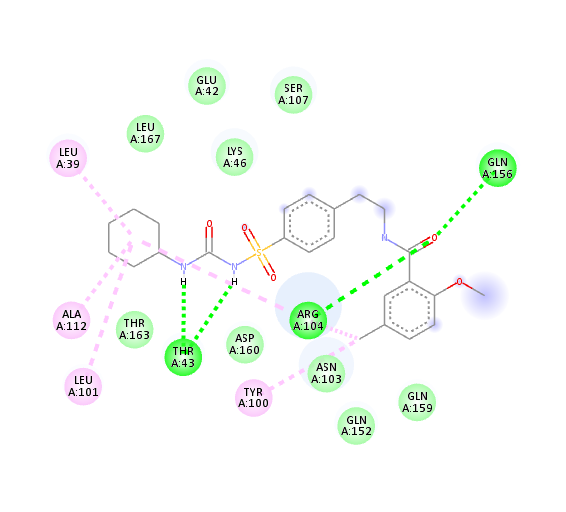** | **d) 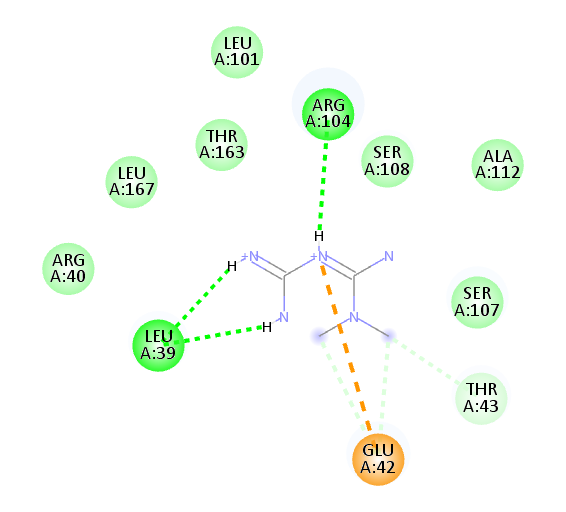** |

**Figure S3:** 2D representation of (a) 9-Oxabicyclo[3.3.1]nonane-2,6-diol, (b) 1,2,3-Benzenetriol, (c) Glibenclamide and (d) Metformin interactions in the binding pocket of IL-6. Green, light blue, pink, orange and violet broken lines represent conventional hydrogen, carbon-hydrogen, pi-alkyl, attractive and pi-sigma bonds, respectively. Light green circles represent Van der Waals interactions.

| **a) 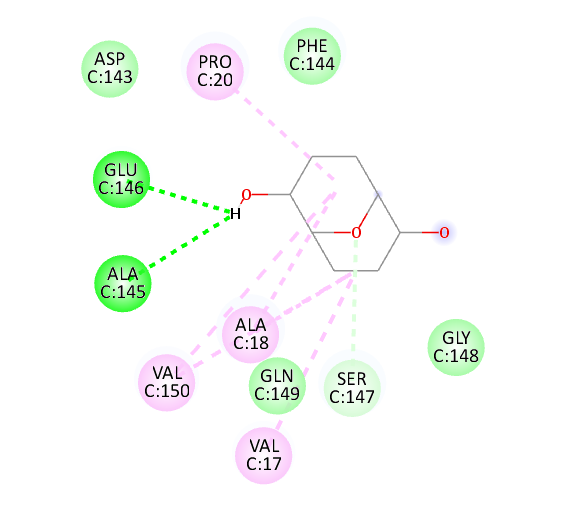** | **b) 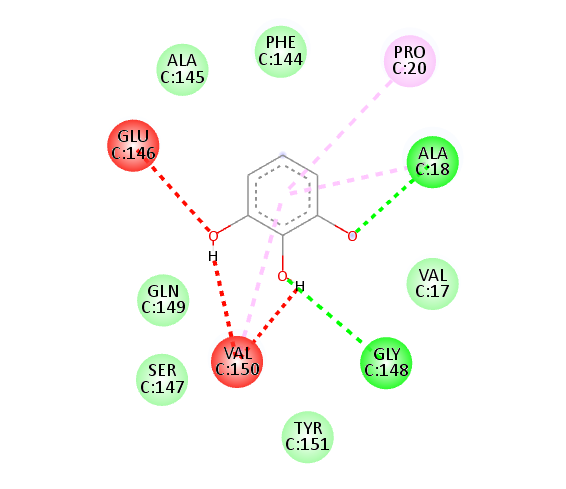** |
| --- | --- |
| **c) 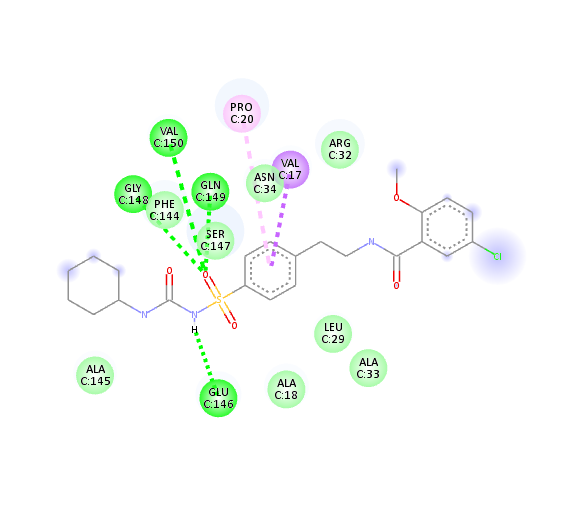** | **d) 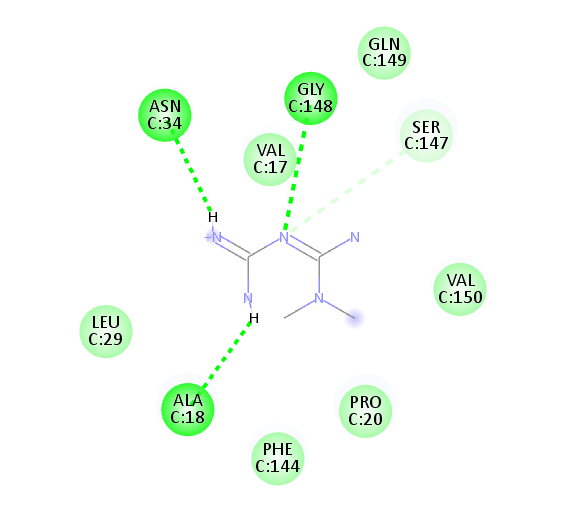** |

**Figure S4:** 2D representation of (a) 9-Oxabicyclo[3.3.1]nonane-2,6-diol, (b) 1,2,3-Benzenetriol, (c) Glibenclamide and (d) Metformin interactions in the binding pocket of TNF-α. Green, light blue, pink, violet and broken red lines represent conventional hydrogen, carbon-hydrogen, pi-alkyl, pi-sigma and unfavourable bonds, respectively. Light green circles represent Van der Waals interactions.
